# Prevalence of weight excess according to age group in students from Campinas, SP, Brazil

**DOI:** 10.1590/0103-0582201432214713

**Published:** 2014-06

**Authors:** Silvia Diez Castilho, Luciana Bertoldi Nucci, Lucca Ortolan Hansen, Samanta Ramos Assuino

**Affiliations:** 1 Faculdade de Medicina da PUC-Campinas, Campinas, SP, Brasil

**Keywords:** body mass index, child, adolescent, overweight, obesity

## Abstract

**OBJECTIVE:**

To evaluate the prevalence of weight excess in children and adolescents attending public and private schools of Campinas, Southeast Brazil, according to age group.

**METHODS:**

Cross-sectional study that enrolled 3,130 students from 2010 to 2012. The weight and the height were measured and the body mass index (BMI) was calculated. The students were classified by BMI Z-score/age curves of the World Health Organization (WHO)-2007 (thinness, normal weight, overweight and obesity) and by age group (7-10, 11-14 and 15-18 years). Multinomial logistic regression analysis was applied to verify variables associated to overweight and obesity.

**RESULTS:**

Among the 3,130 students, 53.7% attended public schools and 53.4% were girls. The prevalence of weight excess (overweight or obesity) was higher in private schools (37.3%) than in public ones (32.9%) and among males (37.5%), compared to females (32.7%; *p*<0.05). The chance of having weight excess in children aged 7-10 years was more than twice of those over 15 years old (OR 2.4; 95%CI 2.0-3.0) and it was 60% higher for the group with 11-14 years old (OR 1.6; 95%CI 1.3-2.0). The chance of being obese was three times higher in 7-10 years old children than in the adolescents with 15-18 years old (OR 4.4; 95%CI 3.3-6.4) and 130% higher than the group with 11-14 years old (OR 2.3; 95%CI 1.6-3.2).

**CONCLUSIONS:**

The prevalence of weight excess in Campinas keeps increasing at an alarming rate, especially in the younger age group.

## Abstract (es)

**OBJETIVO:**

Evaluar la prevalencia de exceso de peso en niños y adolescentes que estudian en escuelas privadas y públicas de Campinas, São Paulo, conforme a la franja de edad.

**MÉTODOS:**

Estudio transversal que evaluó a 3.130 alumnos de 2010 a 2012, con medición de peso y estatura y cálculo del índice de masa corporal (IMC). Los alumnos fueron clasificados por el escore z del IMC/edad de las curvas de la Organización Mundial de la Salud (OMS)-2007 (delgadez, eutrofia, sobrepeso y obesidad) y franja de edad (7-10, 11-14 y 15-18 años). Se aplicó el análisis de regresión logístico multinomial para evaluar los factores asociados al sobrepeso y a la obesidad.

**RESULTADOS:**

De los 3.130 alumnos, el 53,7% frecuentaba escuelas públicas y el 53,4% eran muchachas. La prevalencia de exceso de peso (sobrepeso u obesidad) fue mayor en las escuelas privadas (37,3%) que en las públicas (32,9%) y en los muchachos (37,5%), si comparados a las muchachas (32,7%; *p*<0,05). La posibilidad de exceso de peso en los niños con 7-10 años fue más que el doble de la que de adolescentes con 15 años o más (OR 2,4; IC95% 2,0-3,0) y, para el grupo de 11-14 años, fue el 60% más grande (OR 1,6; IC95% 1,3-2,0). La posibilidad de ser obeso, a su vez, en los niños de 7-10 años fue más que el triple de la que en adolescentes de 15-18 años (OR 4,4; IC95% 3,3-6,4) y el 130% mayor que la de adolescentes de 11-14 años (OR 2,3; IC95% 1,6-3,2).

**CONCLUSIONES:**

La prevalencia del exceso de peso en Campinas sigue aumentando de modo alarmante, principalmente en la franja de edad más joven.

## Introduction

Even though some countries such as the United States, Sweden, Switzerland, France, and Australia have been containing or even reversing, albeit timidly, the increase of obesity prevalence in determined age groups^(^ 1 ^,^ 2 ^)^, data from the Household Budget Survey (*Pesquisa de Orçamentos Familiares* - POF) 2008-2009 show that, in the Brazilian population, it is increasing in all age groups and social classes^(^ 3 ^)^. The trend towards the increase of excess weight (overweight + obesity) observed between 1974/1975 and 2008/2009 was higher among male adolescents between 10 and 19 years (4.1 to 27.6%), while in 2008-2009, there was higher prevalence between children from 5 to 9 (51.4% in boys and 43.8% in girls)^(^ 3 ^)^. 

In 2011, the Lancet magazine has published a series that examines the global escalade of the obesity epidemics observed in the last 4 decades. The articles discuss the trend of this pandemy^(^ 2 ^)^ and its economic consequences, as it reflects on the morbidity and mortality of the population, decreasing productivity and increasing public expenses with health care^(^ 4 ^)^. They also discuss aspects of control and maintenance of weight^(^ 5 ^)^ and actions necessary to contain the current obesogenic environment and reverse risk factors for chronic diseases^(^ 6 ^)^. The Brazilian government has been adopting various measures for years to contain the epidemics in our country, and to fight especially child obesity^(^ 7 ^)^. Although the resolution of the problem is quite complex, these measures have, in general, tried to encompass the directives pointed by the specialists^(^ 2 ^,^ 6 ^)^. It is necessary, however, to verify the effectiveness of these actions.

The assessment of prevalence of excess weight according to the age range in school children may indicate the trend to be observed within the next years. Therefore, this study aimed to describe the prevalence of excess weight among children and adolescents from Campinas, state of São Paulo, according to the age group. 

## Method

Data analyzed in this study are part of two cross-sectional samples collected in private and public (state) schools from Campinas, between 2010 and 2012. According to the school census from 2011^(^ 8 ^)^, a year that is intermediate to those of the samples, the estimated population in the age range enrolled in these schools (236 private and 491 public) was of 133,087 students. The included schools were selected through random draw, among all school within the municipality that had students in the studied age range. The sorting order was respected, and of eight private schools, five agreed to participate in the study. Among state schools, 15 schools were contacted and eight participated. This sequence was repeated until we reached the number of subjects planned. We assessed the students who had the consent of the principals, parents, or guardians, and those who, at the time of data collection, agreed to participate. 

Among the 3,178 assessed students, 3,130 students were included, from both sexes, and in the age range from 7 to 18 years (<19 years), who did not present conditions such as pregnancy, plaster splints, being a wheelchair user, uncorrected disease, or use of medications that could impair the assessment of weight and height gain or measurements. We excluded 48 students who did not meet the inclusion criteria. The sample was initially calculated to assess the body mass index (BMI) according to Tanner maturation stages and the calculations of its size are properly described in previous studies^(^ 9 ^,^ 10 ^)^. However, we considered the sample size adequate for a prevalence estimate, once the calculation - using as parameters a proportion of 50% of overweight in the population, sampling error of 2%, and a confidence level of 95% - was of 2,376 students.

Height and weight were obtained (Wiso^(r)^ wall anthropometer and Tanita^(r)^ scale) by the same researcher, according to international standards^(^ 11 ^)^, and the BMI was calculated. Students were classified by BMI/age Z score curves of the World Health Organization (WHO)-2007 (thinness, eutrophy, overweight, and obesity) for the analyzed age groups (7-10, 11-14 and 15-18 years), being subsequently grouped according to BMI in: excess weight (overweight + obesity) and without excess weight (thinness + eutrophy).

Analyses were performed using the Statistical Package for the Social Sciences (SPSS) v.17.0 (SPSS Inc., Chicago, USA), using Student *t* test to compare means, chi-square test to compare proportions, and multinomial logistic regression analysis to verify the factors associated with overweight and obesity, with a significance level of 5%. 

To discuss the policies that have been adopted in Brazil to control the weight gain trend in the population, we carried out a search in the Google database and Google Scholar, with a limit of 15 years.

This study was approved by the Research Ethics Committee of the Institution (Pontifical Catholic University of Campinas - PUC-Campinas - protocol n. 693/09 and 574/11).

## Results

Among the 3,130 students assessed, 1,450 (46.3%) attended private schools and 1,680 (53.7%), public; 1,671 (53.4%) were girls and 1,459 (46.6%) boys. The prevalence of excess weight was higher in private schools than in public schools, and higher among boys, compared to girls (Table 1).

**Table 1:**
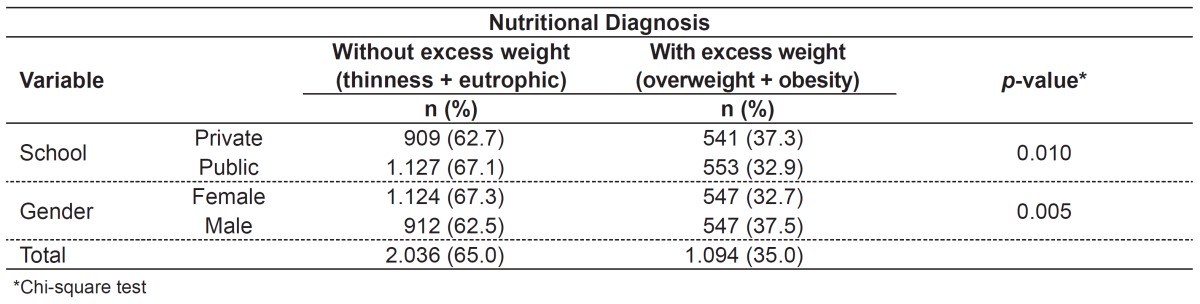
Nutritional diagnosis of students assessed in Campinas, SP (2010-2012), according to type of school and gender

The mean age (years) was lower in children with excess weight (11.9±2.9) if compared to normal weight children (12.8±3.1; *p*<0.001). Excess weight in the sample was more prevalent among younger students: 43.5% of children from 7-10 years, 33.8% of adolescents between 11-14 years, and 24.5% of those older than 15 years. 

Table 2 shows the nutritional assessment of students and the chance of being overweight and obese according to age, adjusted for school type and gender. The chance of having excess weight (overweight + obesity) in children with 7-10 was more than double compared to the adolescents aged 15 years or more (OR 2.4; 95%CI 2.0-3.0; *p*<0.001) and, for the group of 11-14 years, the chance was 60% higher (OR 1.6; 95%CI 1.3-2.0; *p*<0.001). The chance of being obese among children from 7-10 years was more than triple compared to adolescents aged 15 and 18 years and 130% greater than that found in the age group 11-14 years.

**Table 2:**
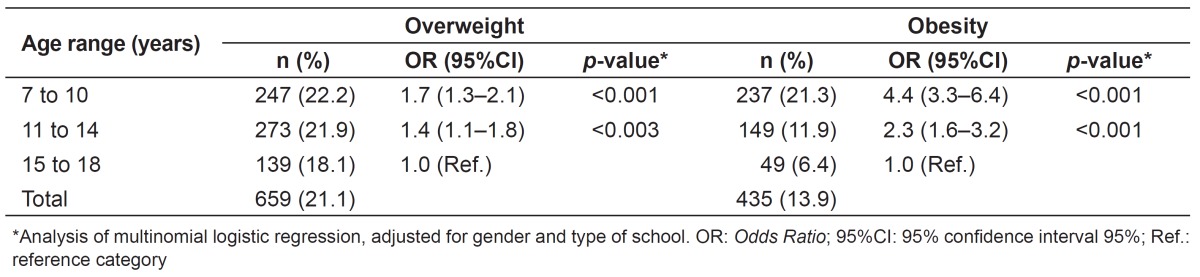
Prevalence (%) of overweight and obesity in 3,130 students assessed and the respective odds ratio according to age range. Campinas, SP (2010-2012)

## Discussion

The stratification of children by age group allows observing the age range in which excess weight predominates. It is noteworthy that the prevalence is much higher among younger students. The results of this study also show that the problem is more frequent in students from private schools and among boys. In 4 years, these children are going to compose the subsequent age stratum, which may point to an increase in the prevalence of excess weight in the country, in case their BMI does not decrease with the growth spurt of puberty. The population will be increasingly obese, and, consequently, at greater risk of having health and productivity compromised, bringing an increasing burden on the country's economy.

In recent decades, Brazil has undergone a nutritional transition characterized by the decline of malnutrition and the increase in overweight and obesity. The change in the nutritional profile is due to urbanization and industrialization, that lead to an increase in caloric intake and decrease in physical activity, with consequent accumulation of fat^(^ 12 ^)^. Weaning and early introduction of high-calorie processed foods, besides the reduced space for physical exercise and the incorporation of forms of sedentary leisure, complete this scenario^(^ 13 ^)^. This transition has been faster than in other countries undergoing similar processes, such as China^(^ 14 ^)^. This can be explained by the fact that habits and feeding practices are constructed based on sociocultural determinants. In this respect, the media, through marketing campaigns, plays an important role in the construction and deconstruction of these practices^(^ 12 ^)^. Other facts that contribute to this scenario are women's work outside the home - which influences the food purchase profile, food preparation, and consumption of meals outside the home -, as well as the form of production, supply, distribution, market control, and consumption of food, based on technological, economic, and social changes which the country has undergone^(^ 12 ^,^ 13 ^)^. 

A few decades ago, the Brazilian government has taken a series of measures to tackle these nutritional problems, which include not only malnutrition and deficiency diseases related to malnutrition, but also obesity^(^ 7 ^)^. The National School Meal Program (*Programa Nacional de Alimentação Escolar* - PNAE), developed in 1954 and which, initially, was directed to schools in the Northeast to reduce malnutrition, gradually gained nationwide coverage. School feeding has become a constitutional right in 1988. In 1994, PNAE was decentralized (Law n. 8.913), when the federal government established a partnership with states and municipalities to transfer exclusive financial resources for the purchase of food. From that date on, the states and municipalities were in charge of preparing the menu, purchasing food, performing quality control, hiring human resources, and providing adequate infrastructure for the provision of school meals, and it was necessary to complement the funds allocated by the Federal Government with additional resources. In 1999, the decree n. 710/1999, established the National Food and Nutrition Policy (*Política Nacional de Alimentação e Nutrição* - PNAN), determining actions to provide universal access to food, besides ensuring their safety and quality, monitoring the food and nutrition conditions, promoting healthy feeding practices and lifestyles, preventing and controlling nutritional disorders, promoting the development of health care, and empowering human resources directed to health and nutrition^(^ 15 ^)^. 

In 2006, the Brazilian Ministry of Health prepared guidelines^(^ 16 ^)^ to provide the adhesion of the school community to healthy eating habits for health promotion, within the program "Promoting Healthy Eating at School" (*Promoção da Alimentação Saudável na Escola)* ^(^ 17 ^)^ - for preschool, elementary, and high school in the public and private sectors (ordinance n. 1.010/2006). This program, in addition to encouraging the establishment of orchards, regulates the trade and preparation of foods high in fat, sugar, and salt in the school environment, and encourages the consumption of fruits and vegetables. In the following year, the School Health Program (*Programa Saúde na Escola* - PSE), was created by the presidencial decree n. 6.286/2007^(^ 18 ^)^, with the aim to ensure access to a diet with a minimum daily nutritional intake that would enable an improved health and academic performance of public schools students, and to encourage healthy feeding habits among the beneficiaries. Some proposals have been sitting in Congress for years, such as the project n. 5.921/2001, trying to regulate the advertising of unhealthy foods conveyed in the media (radio, television and internet) between 7a.m and 9p.m and outside these hours, only with warnings on the damage to health^(^ 7 ^)^. Recently the resolution n. 24/2010 by the National Health Surveillance Agency (*Agência Nacional de Vigilância Sanitária* - Anvisa) legislated on the theme to prevent the increased risks of non-communicable chronic diseases in the more vulnerable public, especially children and adolescents^(^ 19 ^)^. 

These interventions attempt to integrate actions to encourage, support, and protect health. In 2012, the National Food Security Council (*Conselho Nacional de Segurança Alimentar* - Consea) discussed the Intersectoral Plan for the Prevention and Control of Obesity^(^ 20 ^)^, proposed by the Interministerial Chamber on Food Security and Nutrition (*Câmara Interministerial de Segurança Alimentar e Nutricional* - Caisan), and concluded that the prevalence of obesity imposes significant challenges to the state and society. This plan innovates when starts to consider that obesity is not only the responsibility of the citizen or the health and education sectors, but reflects the way society is organized, produces food, affects the consumption, and transforms goods into commodities^(^ 20 ^,^ 21 ^)^. Thus, the solution of the problem becomes intersectoral, and no longer a medical and personal condition, as the search for solutions must now involve both the government and society. 

In July 2012, São Paulo approved the law n. 14.830, according to the bill 369/2011, which created the National Program in pubic schools to prevent obesity^(^ 22 ^)^. This law determines the hiring of nutritionists to develop a menu of meals offered in state schools and the availability of pediatricians to follow the evolution of children's weight. 

In the municipality of Campinas, state of São Paulo, where the sample was evaluated, the school snacks are served in 100% of the 491 public schools. Among the 236 private schools, 85 (36%) provide meals to be purchased by students, according to the school census of 2011^(^ 8 ^)^. The program that takes care of feeding in public schools is managed by the Municipal Education Secretariat and operated by the Central Food Supply of Campinas (Ceasa) since 2002, by nutritionists who prepare menus to meet the nutritional needs of the students, (15% of daily need for students who are in school part-time and 75% for those attending full-time). In total, 16 menus are prepared daily to contemplate the different age ranges. Students with health problems (diabetes, intolerance or food allergy) are also catered for, and their food is prepared according to their needs. Thus, the municipality started to rely on the work of nutritionists 10 years before the (state) law that established the hiring of these professionals. The purchase of the products listed is made by the municipality through public bids. The food is then distributed to schools, where trained cooks prepare the pre-designed menus. The meals offered in the municipality are among the top-rated in the country^(^ 23 ^)^. Even in these schools, there are canteens that sell treats, (chocolates, candies, snacks, etc.), soft drinks, and fried snacks in between, which favors the consumption of unhealthy foods. 

In the private schools of Campinas, the consumption of sweets is also common. Although these students - who normally attend school part-time (morning or afternoon) - can bring snacks from home, there is a preference for food purchased in the cafeteria. In some private schools, the cafeteria offers meals that may be purchased if the child has some extracurricular activity, but there is no information on compliance with the law regarding the hiring of nutritionists to guide menus. 

The advertising of unhealthy foods on children's TV programs, as well as the offer of advantages in the purchase of determined foods (such as offering toys inside the package or at the purchase of snacks), physical activity limited by urban violence, and the shortage of areas for leisure and sporting activities - despite government initiatives - still influence the bad habits that promote weight gain in the municipality of Campinas. Actions to encourage the practice of exercises are more common in the private schools analyzed, which organize tournaments to stimulate physical activity. Therefore, we concluded that the solution of the problem is complex.

For the objectives of fighting overweight to be achieved, it is necessary that the government not only lead actions, create laws, allocate resources, but also make ensure that these initiatives be met, to guarantee a healthy environment as well as facilitators for weight loss and maintenance in the long term. At the same time, it is up to the citizen (family and community) to follow guidelines, choosing and preparing healthy meals, and practicing physical activities at the locations provided by public organs. 

In 2009, the National Survey of School Health (*Pesquisa Nacional de Saúde Escolar* - *PeNSE*)^(^ 24 ^)^ assessed students from public and private schools from all over the country. This research, which included, among other things, questions about diet, physical activity, and time spent in front of the TV, shows that more than half of the students do not practice exercises, with percentages ranging from 65.8 to 49%. The research also found that students from public schools are more inactive (57.4%) than those from private schools (54.9%) in the Federal District, and that the consumption of sweets (50.9%) in five or more days of the week exceeds that of fruit (31.5%) and vegetables (32,4%). Regarding time watching television, 80% spent 2 or more hours in front of the screen, while the WHO recommends a maximum of 2 hours.

In this study, we did not collect any data on nutrition, physical activity, or data related to sedentary activities, such as time spent in front of the television or the computer. However, all public schools provided at least two meals per day (a snack and a meal, for those attending part-time). In the case of children with no resources to receive an adequate nutrition at home (breakfast, two salty meals, and two snacks), the meal eaten at school must ensure at least the minimum daily intake, but, for others, it represents a third salty meal, which may contribute to weight gain. It is noteworthy that, even with a smaller prevalence of overweight among students from public schools (32.9%) if compared to those of private schools (37.3%), it is more than double the expected for the general population (15% overweight, being 12% overweight children, and 3% obese children). Another issue already discussed is the fact that many children do not eat school snacks, preferring the snack brought from home or that sold in the canteen^(^ 7 ^)^. 

Regarding the prevalence of excess weight according to sex, there was a predominance prevalence in boys (37.5%) compared to girls (32.7%), the data concerning children and adolescents between 7 and 18 years are in accordance with the results by the Household Budget Survey 2008-2009^(^ 25 ^)^, both for children from 5 to 9 and adolescents between 10 and 19. In these age ranges, the boys presented a higher prevalence of overweight compared to girls.

It is noteworthy that, despite the government initiatives, progress on tackling overweight has been small, since children are increasingly obese. Many of the initiatives are only regulations, resolutions, and decrees, which do not have the strength of law, and function only as recommendations. Other proposals have been sitting in Congress for years, where diverse interests of certain sectors, such as the processed food industry, are working against its approval. The consensus is that isolated actions are not enough to stop this advance, as "the increase in weight results from a normal response, of normal people, to an abnormal environment"^(^ 21 ^)^. 

The publication of the POF 2008-2009 considers that the high prevalence of obesity calls for the implementation of new steps to control it, including tax policies that increase people's access to healthy foods and intervention in the public space aiming to the practice of physical activity^(^ 24 ^)^. Plans, recommendations, and even some laws do exist but need to be respected. There is no way to address the problem without the involvement of all sectors of society^(^ 26 ^)^. It is up to the government to provide favorable conditions, however, without the help of the school and the family, and without the individual's self-will, it becomes difficult to achieve the desired goal.

There is no need to wait for a new research by the Household Budget Survey to realize that the measures taken so far have not produced the desired effect in the sample, since the prevalence remains high in all age groups analyzed. It is important to track down the trend of overweight, but it is also important to learn from the experience of countries that have succeeded in containing it. 
