# Supplementary material for: Prevalence of weight excess according to age group in students from Campinas, SP, Brazil
Source: Rev Paul Pediatr. 2014 Jun;32(2):200–6. doi: 10.1590/0103-0582201432214713 (PMC4183029; doi:10.1590/0103-0582201432214713)
Supplement: Translation

# Translation: Prevalência de excesso de peso conforme a faixa etária em alunos de escolas de Campinas, SP

**Authors:** Silvia Diez Castilho, Luciana Bertoldi Nucci, Lucca Ortolan Hansen, Samanta Ramos Assuino

**Affiliations:** 1 Faculdade de Medicina da PUC-Campinas, Campinas, SP, Brasil

**Keywords:** índice de massa corporal, criança, adolescente, sobrepeso, obesidade

## Abstract

**OBJETIVO:**

Avaliar a prevalência de excesso de peso em crianças e adolescentes que estudam em escolas particulares e públicas de Campinas, SP, conforme a faixa etária.

**MÉTODOS:**

Estudo transversal que avaliou 3.130 alunos de 2010 a 2012, com mensuração de peso e estatura e cálculo do índice de massa corpórea (IMC). Classificaram-se os alunos pelo escore Z do IMC/idade das curvas da Organização Mundial da Saúde (OMS)-2007 (magreza, eutrofia, sobrepeso e obesidade) e faixa etária (7-10, 11-14 e 15-18 anos). Aplicou-se a análise de regressão logística multinomial para avaliar os fatores associados ao sobrepeso e à obesidade.

**RESULTADOS:**

Dos 3.130 alunos, 53,7% frequentavam escolas públicas e 53,4% eram meninas. A prevalência de excesso de peso (sobrepeso ou obesidade) foi maior nas escolas privadas (37,3%) do que nas públicas (32,9%) e nos meninos (37,5%), se comparados às meninas (32,7%; *p*<0,05). A chance de ter excesso de peso nas crianças com 7-10 anos foi mais que o dobro do que a de adolescentes com 15 anos ou mais (OR 2,4; IC95% 2,0-3,0) e, para o grupo de 11-14 anos, foi 60% maior (OR 1,6; IC95% 1,3-2,0). Já a chance de ser obeso nas crianças com 7-10 anos foi mais que o triplo do que a de adolescentes de 15-18 anos (OR 4,4; IC95% 3,3-6,4) e 130% maior do que a de adolescentes de 11-14 anos (OR 2,3; IC95% 1,6-3,2).

**CONCLUSÕES:**

A prevalência do excesso de peso em Campinas continua aumentando de forma alarmante, principalmente na faixa etária mais jovem.

## Introdução

Embora alguns poucos países como Estados Unidos, Suécia, Suíça, França e Austrália estejam conseguindo conter ou até reverter, ainda que de modo tímido, o aumento da prevalência da obesidade em determinadas faixas etárias^(^ ^1^ ^,^ ^2^ ^)^, dados da Pesquisa de Orçamentos Familiares (POF) 2008-2009 mostram que, na população brasileira, ela está aumentando em todas as idades e classes sociais^(^ ^3^ ^)^. A tendência ao aumento do excesso de peso (sobrepeso + obesidade) observada entre 1974/1975 e 2008/2009 foi maior entre adolescentes de dez a 19 anos do sexo masculino (4,1 para 27,6%), embora tenha sido observada, em 2008-2009, maior prevalência entre as crianças de cinco a nove anos (51,4% nos meninos e 43,8% nas meninas)^(^ ^3^ ^)^. 

Em 2011, a revista Lancet publicou uma série que examina a escalada global da epidemia de obesidade observada nas últimas quatro décadas. Os artigos discutem a tendência dessa pandemia^(^ ^2^ ^)^ e suas consequências econômicas, à medida que repercute sobre a morbimortalidade da população, diminuindo a produtividade e aumentando os gastos públicos com a saúde^(^ ^4^ ^)^. Também discutem aspectos do controle e manutenção de peso^(^ ^5^ ^)^ e ações necessárias para conter o atual ambiente obesogênico e reverter os fatores de risco para doenças crônicas^(^ ^6^ ^)^. O governo brasileiro vem adotando várias medidas, há anos, para conter a epidemia em nosso meio e combater principalmente a obesidade infantil^(^ ^7^ ^)^. Embora a resolução do problema seja bastante complexa, essas medidas têm, de modo geral, tentado abranger as diretrizes apontadas pelos especialistas como necessárias^(^ ^2^ ^,^ ^6^ ^)^. É preciso, no entanto, verificar a efetividade dessas ações.

A avaliação da prevalência do excesso de peso segundo a faixa etária em escolares pode indicar a tendência a ser observada nos próximos anos. Portanto, o objetivo deste estudo foi descrever a prevalência de excesso de peso em crianças e adolescentes de Campinas, SP, conforme a faixa etária. 

## Método

Os dados analisados neste estudo fazem parte de duas amostras transversais colhidas em escolas particulares e públicas (estaduais) de Campinas, SP, entre 2010 e 2012. De acordo com o censo escolar 2011^(^ ^8^ ^)^, ano intermediário às amostras, a população estimada na faixa etária estudada matriculada nessas escolas (236 particulares e 491 públicas) era de 133.087 alunos. As escolas incluídas foram selecionadas mediante sorteio aleatório, entre todas as escolas do município que possuíam alunos na faixa etária de interesse. A ordem de sorteio foi respeitada, sendo que, das oito escolas particulares, cinco aceitam participar. Entre as públicas (estaduais), 15 escolas foram contatadas e oito participaram. Essa sequência foi seguida até se atingir o número de sujeitos previstos. Avaliaram-se os alunos que tiveram o consentimento dos diretores e pais ou responsáveis e que, no momento da coleta de dados, assentiram em participar. 

Dos 3.178 avaliados, incluíram-se 3.130 alunos de ambos os sexos e na faixa etária de sete a 18 anos (<19 anos), que não apresentavam condição como gravidez, tala gessada, ser cadeirante, doença não corrigida ou uso de medicação que prejudicasse a avaliação do ganho de peso e estatura ou a tomada das medidas. Excluíram-se 48 alunos que não preencheram os critérios de inclusão. A amostra foi inicialmente calculada para avaliar o índice de massa corpórea (IMC) conforme os estágios maturação de Tanner e os cálculos de seu tamanho estão devidamente descritos em estudos já publicados^(^ ^9^ ^,^ ^10^ ^)^. Entretanto, considerou-se o tamanho da amostra adequado para uma estimativa de prevalência, uma vez que o cálculo - utilizando-se como parâmetros uma proporção de 50% de excesso de peso na população, erro amostral de 2% e nível de confiança de 95% - foi de 2.376 alunos.

A estatura e o peso foram obtidos (antropômetro de parede Wiso^(r)^ e balança Tanita^(r))^ pela mesma pesquisadora segundo as normas internacionais^(^ ^11^ ^)^ e o IMC foi calculado. Classificaram-se os alunos pelo escore Z do IMC/idade das curvas da Organização Mundial da Saúde (OMS) - 2007 (magreza, eutrofia, sobrepeso e obesidade) para as faixas etárias analisadas (7-10, 11-14 e 15-18 anos), sendo posteriormente agrupados conforme o IMC em: com excesso de peso (sobrepeso + obesidade) e sem excesso de peso (magreza + eutrofia).

As análises foram realizadas no programa *Statistical Package for the Social Sciences* (SPSS) v.17.0 (SPSS Inc., Chicago, USA), utilizando-se o teste *t* de Student para comparar médias, o teste do qui-quadrado para comparar proporções e a análise de regressão logística multinomial para verificar os fatores associados a sobrepeso e obesidade, com um nível de significância de 5%. 

Para discutir as políticas que têm sido adotadas no Brasil para controlar a tendência ao aumento de peso na população, realizou-se uma busca em banco de dados do Google e Google Acadêmico, com limite dos últimos 15 anos.

Esta pesquisa foi aprovada pelo Comitê de Ética em Pesquisa da Pontifícia Universidade Católica de Campinas.

## Resultados

Dos 3.130 alunos avaliados, 1.450 (46,3%) frequentavam escolas particulares e 1.680 (53,7%), públicas; 1.671 (53,4%) eram meninas e 1.459 (46,6%) meninos. A prevalência de excesso de peso foi maior nas escolas particulares do que nas públicas e entre os meninos, se comparados às meninas (Tabela 1).

**Tabela 1:** Diagnóstico nutricional dos alunos avaliados em Campinas, SP (2010-2012), conforme o tipo de escola e sexo

A média de idade (anos) foi menor nas crianças com excesso de peso (11,9±2,9) se comparada às sem excesso de peso (12,8±3,1; *p*<0,001). O excesso de peso na amostra foi mais prevalente entre os alunos mais jovens: 43,5% das crianças de 7-10 anos, 33,8% dos adolescentes entre 11-14 anos e 24,5% dos maiores de 15 anos. 

A Tabela 2 mostra o diagnóstico nutricional dos alunos e a chance de apresentar sobrepeso e obesidade conforme a faixa etária, ajustada por tipo de escola e sexo. A chance de ter excesso de peso (sobrepeso + obesidade) nas crianças com 7-10 anos foi mais que o dobro se comparada à de adolescentes com 15 anos ou mais (OR 2,4; IC95% 2,0-3,0; *p*<0,001) e, para o grupo de 11-14 anos, a chance foi 60% maior (OR 1,6; IC95% 1,3-2,0; *p*<0,001). A chance de ser obeso entre as crianças com 7-10 anos foi mais que o triplo se comparada à de adolescentes com idades entre 15 e 18 anos e 130% maior que a do grupo de 11-14 anos.

**Tabela 2:** Prevalência (%) de sobrepeso e obesidade em 3.130 alunos avaliados e a respectiva razão de chance, conforme a faixa etária. Campinas, SP (2010-2012)

## Discussão

A estratificação das crianças por grupo etário permite observar qual a faixa etária em que predomina o excesso de peso. Nota-se que a prevalência é bem maior entre os estudantes mais jovens. Os resultados deste estudo mostram ainda que o problema é mais frequente nos alunos de escolas particulares e entre os meninos. Em quatro anos, essas crianças irão compor o estrato etário subsequente, o que pode apontar para um aumento da prevalência do excesso de peso em nosso meio, caso seu IMC não diminua com o estirão da puberdade. A população estará cada vez mais obesa e, consequentemente, com maior risco de ter sua saúde e produtividade comprometidas, trazendo um ônus cada vez maior à economia do país.

Nas últimas décadas, o Brasil tem passado por uma transição nutricional caracterizada pelo declínio da desnutrição e pelo aumento do sobrepeso e da obesidade. A mudança do perfil nutricional decorre da urbanização e da industrialização, que levam ao aumento da ingestão de calorias e à diminuição da atividade física, com consequente acúmulo de gordura^(^ ^12^ ^)^. O desmame e a introdução precoce de alimentos processados altamente calóricos, além da diminuição do espaço para a prática de exercícios físicos e a incorporação de formas de lazer sedentárias, completam esse cenário^(^ ^13^ ^)^. Essa transição tem sido mais rápida do que em outros países que passam por processo similar, como no caso da China^(^ ^14^ ^)^. Isso pode ser explicado pelo fato de os hábitos e práticas alimentares serem construídos com base em determinantes socioculturais. Nesse aspecto, a mídia, por meio das campanhas de *marketing*, desempenha um papel importante na construção e desconstrução dessas práticas^(^ ^12^ ^)^. Contribuem ainda para esse cenário o trabalho da mulher fora de casa - que acaba influenciando no perfil de compra de alimentos, no seu preparo e no consumo de refeições servidas fora do domicílio -, bem como a forma de produção, abastecimento, distribuição, controle de mercado e consumo de alimentos, pautadas nas mudanças tecnológicas, econômicas e sociais pelas quais o país tem passado^(^ ^12^ ^,^ ^13^ ^)^. 

Há algumas décadas, o governo brasileiro vem tomando uma série de medidas para lidar com esses problemas nutricionais, que compreendem não só a desnutrição e as doenças carenciais ligadas à má nutrição, mas também a obesidade^(^ ^7^ ^)^. O Programa Nacional de Alimentação Escolar (PNAE), desenvolvido a partir de 1954 e que, inicialmente, atendia escolas do Nordeste para reduzir a desnutrição, aos poucos ganhou abrangência nacional. A alimentação escolar passou a ser um direito constitucional em 1988. Em 1994, instituiu-se a descentralização do PNAE (lei nº 8.913), quando o governo federal estabeleceu convênio com estados e municípios para o repasse de recursos financeiros exclusivos para a aquisição de alimentos. A partir dessa data, passou a ficar a cargo dos estados e municípios elaborar o cardápio, adquirir alimentos, realizar o controle de qualidade, contratar recursos humanos e providenciar a infraestrutura adequada para o fornecimento da merenda escolar, sendo necessário complementar com recursos próprios a verba repassada pela União. Em 1999, surgiu a portaria nº 710/1999, que estabeleceu a Política Nacional de Alimentação e Nutrição (PNAN), determinando ações para o acesso universal aos alimentos, além de garantir sua segurança e qualidade, monitorar a situação alimentar e nutricional, promover práticas alimentares e estilos de vida saudáveis, prevenir e controlar os distúrbios nutricionais, promover o desenvolvimento da saúde e capacitar recursos humanos em saúde e nutrição^(^ ^15^ ^)^. 

Em 2006, o Ministério da Saúde elaborou diretrizes^(^ ^16^ ^)^ para propiciar a adesão da comunidade escolar a hábitos alimentares saudáveis para a promoção da saúde, dentro do programa Promoção da Alimentação Saudável na Escola^(^ ^17^ ^)^ - para educação infantil, fundamental e ensino médio das redes públicas e privadas (portaria nº 1.010/2006). Esse programa, além de estimular a criação de hortas, regulamenta o comércio e o preparo de alimentos com altos teores de gordura, açúcar e sal no ambiente escolar e estimula o consumo de frutas e hortaliças. No ano seguinte, criou-se o Programa Saúde na Escola (PSE), pelo decreto presidencial nº 6.286/2007^ (18)^, para garantir o acesso a uma alimentação com oferta nutricional diária mínima que permitisse a melhora da saúde e o desempenho escolar dos alunos de escolas públicas e estimular bons hábitos alimentares entre os beneficiados. Alguns projetos de lei tramitam anos no congresso, a exemplo do projeto nº 5.921/2001, tentando regulamentar a publicidade de alimentos não saudáveis veiculada nos meios de comunicação (rádio, televisão e internet) entre 7h e 21h - fora desses horários, só com advertências sobre os prejuízos à saúde^(^ ^7^ ^)^. Recentemente, a resolução 24/2010 da Agência Nacional de Vigilância Sanitária (Anvisa) dispôs sobre o tema para impedir o aumento do risco de doenças crônicas não transmissíveis no público mais vulnerável, principalmente crianças e adolescentes^(^ ^19^ ^)^. 

Essas intervenções tentam integrar ações de incentivo, apoio e proteção à saúde. Em 2012, o Conselho Nacional de Segurança Alimentar (Consea) discutiu o Plano Intersetorial de Prevenção e Controle da Obesidade^(^ ^20^ ^)^, proposto pela Câmara Interministerial de Segurança Alimentar e Nutricional (Caisan), e concluiu que a prevalência da obesidade em nosso meio impõe significativos desafios ao Estado e à sociedade. Esse plano inova quando passa a considerar que a obesidade não é só responsabilidade do cidadão ou dos setores da saúde e da educação, mas reflete a maneira como a sociedade se organiza, produz alimentos, condiciona o consumo e transforma bens em mercadorias^(^ ^20^ ^,^ ^21^ ^)^. Dessa forma, a solução do problema passa a ser intersetorial, deixando de ser só um problema médico e pessoal, na medida em que a busca de soluções deve envolver tanto o governo como a sociedade. 

No Estado de São Paulo, foi aprovada em 2012 a lei nº 14.830, de 13 de julho de 2012, conforme o projeto de lei 369/2011, que cria o Programa Nutricional nas escolas públicas para prevenir a obesidade^(^ ^22^ ^)^. Essa lei determina a contratação de nutricionistas para elaborar o cardápio das merendas oferecidas nas escolas estaduais e a disponibilidade de médicos pediatras para acompanhar a evolução do peso das crianças. 

Em Campinas, SP, local onde se avaliou a amostra, a merenda escolar é servida em 100% das 491 escolas públicas. Das 236 escolas particulares, 85 (36%) disponibilizam refeições a serem adquiridas pelos alunos, segundo o censo escolar de 2011^(^ ^8^ ^)^. O programa que cuida da alimentação nas escolas públicas é gerenciado pela Secretaria Municipal de Educação e operacionalizado pela Central de Abastecimento de Campinas (Ceasa) desde 2002, por intermédio de nutricionistas que elaboram os cardápios para, com os alimentos disponíveis em cada época do ano, atender às necessidades nutricionais dos alunos (15% das necessidades diárias para alunos que ficam meio período na escola e 75% para os que frequentam período integral). Ao todo, elaboram-se diariamente 16 cardápios para contemplar as diferentes faixas etárias. Alunos com problemas de saúde (diabéticos, os que apresentam intolerância ou alergia alimentar) também são atendidos, sendo sua merenda preparada conforme suas necessidades. Assim, nota-se que o município começou a contar com o trabalho de nutricionistas dez anos antes da lei (estadual) que estabeleceu a obrigatoriedade da contratação desses profissionais. A compra dos produtos indicados é efetuada pela prefeitura por meio de licitações. Os alimentos são então distribuídos para as escolas, onde cozinheiras capacitadas preparam os cardápios previamente elaborados. A merenda oferecida no município está entre as mais bem avaliadas do país^(^ ^23^ ^)^. Mesmo nessas escolas, existem cantinas que vendem guloseimas (chocolates, balas, salgadinhos etc.), refrigerantes e salgados fritos nos intervalos, o que favorece o consumo de alimentos não saudáveis. 

Nas escolas particulares de Campinas, o consumo de guloseimas também é comum. Embora esses alunos - que normalmente frequentam a escola por meio período (manhã ou tarde) - possam levar lanche de casa, observa-se a predileção por ingerirem alimentos comprados na cantina. Em algumas escolas particulares, a cantina disponibiliza refeições que podem ser adquiridas caso a criança tenha alguma atividade extracurricular, mas não há informações sobre o cumprimento da lei quanto à contratação de nutricionistas para orientar os cardápios. 

A propaganda de alimentos não saudáveis na TV em horários de programas infantis, bem como a oferta de vantagens na compra de determinados alimentos (tais como oferecer brinquedos dentro da embalagem ou na aquisição de alimentos) e a atividade física limitada pela violência urbana e pela escassez de áreas para lazer e práticas esportivas, apesar das iniciativas do governo, ainda influenciam os maus hábitos que favorecem o ganho de peso no município de Campinas. Ações para incentivar a prática de exercícios são mais comuns nas escolas particulares avaliadas, que organizam torneios internos para estimular a atividade física. Assim, vê-se que a solução do problema é complexa.

Para que os objetivos do combate ao excesso de peso sejam alcançados, é necessário que o governo não só lidere ações, crie leis, destine recursos, mas também cuide para que essas iniciativas sejam cumpridas, para garantir à população um ambiente e uma alimentação saudáveis e facilitadores para a perda e manutenção do peso em longo prazo. Ao mesmo tempo, cabe ao cidadão (família e comunidade) aproveitar as orientações, escolhendo e preparando refeições saudáveis, e praticar atividades físicas em locais disponibilizados pelos órgãos públicos. 

Em 2009, a Pesquisa Nacional de Saúde Escolar (PeNSE)^(^ ^24^ ^)^ avaliou alunos de escolas públicas e privadas de todo o país. Essa pesquisa, que incluiu, entre outros assuntos, questões sobre alimentação, atividade física e tempo em frente à televisão, mostra que mais da metade dos alunos não pratica exercícios, com variações de 65,8 a 49%, sendo que, no Distrito Federal, os alunos de escolas públicas são mais inativos (57,4%) do que os de escolas particulares (54,9%). O consumo de guloseimas (50,9%) em cinco ou mais dias da semana supera o de frutas (31,5%) e o de hortaliças (32,4%). Quanto ao tempo em frente à televisão, 80% assistem por duas horas ou mais, enquanto a OMS recomenda o máximo de duas horas.

Neste estudo, não foram colhidos dados sobre nutrição, atividade física ou ligados a atividades sedentárias, como o tempo em frente à televisão ou ao computador. No entanto, em todas as escolas públicas, as crianças recebiam pelo menos duas refeições por dia (lanche e uma refeição, para as que frequentam meio período). No caso de crianças sem poder aquisitivo para receber uma alimentação adequada em casa (café da manhã, duas refeições de sal e dois lanches), essa refeição feita na escola deve garantir pelo menos o aporte mínimo nutricional diário, mas, para as outras, representa uma terceira refeição com alimento salgado, o que pode contribuir para o ganho de peso. Nota-se que, mesmo a prevalência do excesso de peso sendo menor entre os alunos de escolas públicas (32,9%), se comparados com os de escolas particulares (37,3%), ela é mais do que o dobro do esperado para a população geral (15% de excesso de peso, sendo 12% de crianças com sobrepeso e 3% de crianças obesas). Outra questão já discutida é o fato de que muitos não consomem a merenda escolar, preferindo o lanche levado de casa ou vendido na cantina^(^ ^7^ ^)^. 

Quanto à prevalência do excesso de peso de acordo com o sexo, com predominância nos meninos (37,5%) em relação às meninas (32,7%), os dados referentes às crianças e adolescentes entre sete e 18 anos estão de acordo com o encontrado na Pesquisa de Orçamentos Familiares (POF) 2008-2009^(^ ^25^ ^)^, tanto para as crianças de cinco a nove anos, quanto para os adolescentes entre dez e 19 anos. Nessas faixas etárias, os meninos apresentam maior prevalência de excesso de peso se comparados às meninas.

Nota-se que, apesar das iniciativas do governo, o progresso quanto ao combate do excesso de peso tem sido pequeno, uma vez que as crianças estão cada vez mais obesas. Muitas das iniciativas não passam de portarias, resoluções e diretrizes, que não têm força de lei e funcionam apenas como recomendações. Outras propostas ficam tramitando por anos no Congresso, onde interesses diversos de determinados setores, tais como os da indústria de alimentos processados, trabalham contra sua aprovação. É consenso que ações isoladas não são suficientes para deter esse avanço, pois "o aumento de peso resulta de uma resposta normal, de pessoas normais, a um ambiente anormal"^(^ ^21^ ^)^. 

A publicação da POF 2008-2009 considera que a alta prevalência da obesidade reclama a implementação de novos passos para seu controle, incluindo políticas fiscais que aumentem o acesso da população a alimentos saudáveis e a intervenção no espaço público visando à prática de atividade física^(^ ^24^ ^)^. Planos, recomendações e até algumas leis existem, mas precisam ser respeitados. Não há como enfrentar o problema sem o envolvimento de todos os setores da sociedade^(^ ^26^ ^)^. Ao governo cabe propiciar condições favoráveis, porém, sem a ajuda da escola, da família e sem a própria vontade do indivíduo, torna-se difícil alcançar o objetivo desejado.

 Não é necessário aguardar nova pesquisa da POF para constatar que as medidas adotadas até o momento não têm surtido o efeito desejado na amostra analisada, pois a prevalência continua alta em todas as faixas etárias analisadas. Rastrear a tendência do excesso de peso é importante, mas é preciso aprender rapidamente com a experiência de países que têm alcançado sucesso para contê-la.
